# Morphological Disparity of the Mouthparts in Polyphagous Species of Largidae (Heteroptera: Pentatomomorpha: Pyrrhocoroidea) Reveals Feeding Specialization

**DOI:** 10.3390/insects11030145

**Published:** 2020-02-26

**Authors:** Yan Wang, Jolanta Brożek, Wu Dai

**Affiliations:** 1 Key Laboratory of Plant Protection Resources and Pest Integrated Management of the Ministry of Education, College of Plant Protection, Northwest A&F University, Yangling 712100, China; wangyan105422@163.com; 2 Faculty of Natural Science, Institute of Biology, Biotechnology and Environmental Protection, University of Silesia in Katowice, Bankowa 9, 40-007 Katowice, Poland; jolanta.brozek@us.edu.pl

**Keywords:** Pyrrhocoroidea, Largidae, mouthparts structures, feeding

## Abstract

Mouthpart structures were observed in four species of Largidae using scanning electron microscopy to investigate their morphological disparity, and linked to changes in feeding specialization. The examined species are pests that feed mainly on seeds and plant sap of forbs, shrubs, and trees. Their external mouthparts are described in detail for the first time herein. The cone-like labrum and four-segmented tube-like labium are shorter in *Physopelta* species than in *Macrocheraia grandis* (Grey). The labium surface in all studied species bears nine types of sensilla (St1-St2, Sb1-3, Sch, Sca1-2, Sm). The distributions of sensilla on particular labial segments varies among the studied species. The tripartite apex of the labium consists of two lateral lobes and an apical plate that is partly divided in *Physopelta* species, and not divided in *Macrocheraia*. Each lateral lobe possesses a sensillar field with 10 thick-walled uniporous sensilla basiconica, one multiporous sensillum styloconicum, and one long non-porous hair sensillum. Each mandibular stylet tip in *M. grandis* has a central tooth placed anteriorly and pairs of teeth arranged dorso-laterally. In *Physopelta*, there are one or two central teeth placed anteriorly but two pairs of teeth dorso-laterally. In all studied species, the inner surfaces of the mandibular stylets have scale-like projections. A left–right asymmetry of the maxillary stylets is noticeable; the external end of the right maxillary stylet is smooth and slightly tapered in *M. grandis* and evidently wider (spoon–like) in the three species of *Physopelta*, while the left end of the stylets is straight and narrow in *M. grandis* in contrast to *Physopelta*, in which the end is straight and wide. No differences in the internal structure of the maxillary stylets were observed among the studied species. Based on structural differences, we inferred that the mandibles and maxillae are more adapted for seed-sucking in *Physopelta* species than in *M. grandis.* *M. grandis* has the ends of the maxillae more narrowed, a trait more adapted for sucking sap from phloem or parenchymal cells.

## 1. Introduction

True bugs (Heteroptera) are a major clade of non-holometabolous insects within the insect order Hemiptera, comprising more than 42,000 described species and exhibiting a vast array of behavioral diversity in terms of feeding and habitat preference [1,2,3]. Diversification of life histories of the Heteroptera began during the Upper Permian, yielding specialized morphological adaptations that enabled these insects to occupy terrestrial and aquatic habitats and to exploit various food sources [4].

Pentatomomorpha is one of the most diverse infraorders of Heteroptera. The great majority of the Pentatomomorpha families (phytophagous group) are plant-feeders, sucking phloem sap or parenchymal cell contents of monocotyledons and dicotyledons, as well as the endosperms of seeds and plant pollen [2,5,6]. The four fairly species-poor early diverging lineages within Heteroptera have retained predatory behaviors [7].

The evolution of feeding strategies in Heteroptera has been the topic of much debate. Cobben [8,9] and Schaefer [10] suggested zoophagy as the original strategy of the group, while Sweet [11] argued that phytophagous forms are the most primitive Heteroptera. The phylogenetic optimization of feeding evolution by Weirauch et al. [7] strongly suggests that the ancestor of Heteroptera was a predator.

In heteropteran bugs, the mouthparts are always composed of the same set of elongated structures that together form a piercing–sucking proboscis. The proboscis consists of the short sclerite of the labrum and epipharynx (the long or short plate of the pharynx continues along the underside of the labrum), a sheath (labium), and piercing stylets. The latter are formed by the mandibles and the maxillae, which are entirely or partly covered by the sheath. Previous studies of hemipteran mouthparts have shown that these structures differ among taxa [8,12,13,14,15,16,17], reflecting adaptations to different diets and the fact that the specialized digestive system has contributed to their success in feeding on a broad range of foods [18]. Previous studies have focused on Pentatomomorpha species of economic importance [19,20]. Seed-feeding pentatomomorphans use mainly a lacerate-and-flush feeding method, but sap–feeding species usually use a stylet-sheath feeding method [21]. Some pentatomomorphan species employ both types of feeding, e.g., in the pentatomid *Palomena angulosa* Motschulsky, phloem feeding is carried out by the stylet-sheath feeding method, and fruit feeding is carried out by lacerate-and-flush feeding method [22]. Numerous seed-sucking species belong to families Largidae and Pyrrhocoridae and to most families of Lygaeoidea. Pyrrhocoridae is a small, economically important family (300 species), the members of which are widely distributed. Several species of *Dysdercus* are major pests of seeds (bolls) of cotton and other Malvaceae. The mouthpart structures were described previously for *Dysdercus fasciatus* by Signoret [23], and for *Pyrrhocoris sibiricus* Kuschakevich by Wang and Dai [17].

The Largidae, a tropicopolitan family, are moderately sized insects ranging from 5 to 16 mm, often with bright and contrasting colors [24]. According to Stehlík [25] 23 valid genera and 220 species-group taxa of Largidae have been recognized. The biology of Largidae is very poorly understood. All species are thought to be phytophagous, either feeding on seeds or plant sap, and they are related to the vegetative parts of forbs, shrubs, and trees [25]. Most species of Largidae are generalists, although a few may take precedence with Euphorbiaceae [23,26,27]. Although Largidae is considered of minor economic importance [28], some of species are of economic impact and may become serious local pests of cotton and other crops. The mouthpart structures and associated sensory organs of Largidae have never been described in detail. The genera of Largidae are quite diversified in their external morphology, but quite uniform in genitalic structures [25]. Therefore, differences in mouthpart structures among species in this group may be useful for taxonomy.

Cobben [8] showed that the right maxillary stylets of phytophagous heteropterans are less deeply serrated than those of predacious heteropterans, and Cohen [12] showed that the direction of barbs in the mandibular stylets varies between the phytophagous and predatory pentatomids. In the latter, barbs are pointed in the direction of the head, while in the phytophagous species, barbs point away from the head.

The essential aims of the present study were to make clear: (1) whether seed-feeding bugs (Largidae: Physopeltinae) show clear modification of the mouthpart structures compared to their hypothetical predaceous ancestors; (2) whether these modifications are characteristic of the two tribes studied; and (3) how the mouthparts of the studied seed-feeding bugs differ from those of sap-feeding phytophagous true bugs (data from references).

The mouthparts of Physopeltini (*Physopelta quadriguttata* Bergroth, *Ph. gutta* (Burmeister), *Ph. cincticallis* Stål), and Lohitini (*Macrocheraia grandis* (Grey)) were investigated with scanning electron microscopy to reveal important characteristics of mouthpart structures, especially the maxillary and mandibular stylets and the distribution of labial sensilla.

Because the few published studies of Pyrrhocoroidea mouthparts have focused on the family Pyrrhocoridae [8,17,29], and no such studies so far have focused on Largidae, we compared the mouthparts of these two families. We expected that the mouthpart structures and feeding behavior observed in individual species would be different due to the use of different host plants and plant tissues.

## 2. Materials and Methods 

### 2.1. Insect Collecting

All Largidae used in this study were collected in China. *Macrocheraia grandis* (Gray) was collected by Du Yimin in Wild Elephant Valley in Xishuangbanna, Yunnan Province (22°10′N, 100°51′E, elev. 747m) in July 2015 and preserved in 95% ethanol. *Physopelta quadriguttata* Bergroth was collected by Zhu Qing in Leigong Mountain, Guizhou Province (26°38′N, 108°21′E, elev. 2080m) in July 2018 and preserved in 95% ethanol. *Physopelta cincticollis* Stål and *Physopelta gutta* (Burmeister) were collected by Wang Yan in Siming Mountain, Ningbo, Zhejiang Province (29°′75N, 121°09′E, elev. 823m) in June 2017.

### 2.2. Samples for Scanning Electron Microscopy (SEM)

Adults were placed in 95% ethanol and cleaned in an ultrasonic cleaner (KQ118, Kunshan, China) for 10s, and rinsed with 95% ethanol several times. The heads were removed with dissecting needles under a stereomicroscope (Olympus SZX10, Tokyo, Japan) and then dehydrated in baths of 100% ethanol twice for 30 min each, before being transferred to a graded series of tert-butyl alcohol (TBA) solutions of 25%, 50%, and 75% (ethanol: TBA was 3:1; 1:1; 1:3), each for 15 min duration, and 100% TBA for 30 min duration. The samples were then placed into a freeze-drier (VFD-21S, SHINKKU VD, Japan) for 3h. The dried specimens were mounted on aluminum stubs using double-sided copper sticky tape and coated with gold/palladium (40/60) in a high-resolution sputter coater (MSP-1S, SHINKKU VD, Tokyo, Japan), and then examined with a T-3400 SEM (Hitachi, Tokyo, Japan) operated at 15 kV or Nova Nano SEM-450 (FEI, Hillsboro, OR, USA) at 5–10 kV. Fifteen individuals of each species were observed.

### 2.3. Image Processing and Morphometric Measurement

Photographs and SEMs were observed and measured after being imported into Adobe Photoshop CS6 (Adobe Systems, San Jose, CA, USA). Statistical analyses were executed using SPSS 19.0 (SPSS, Chicago, IL, USA). Graphs were fitted using Microsoft Office Excel 2007.

### 2.4. Terminology

For main classification of sensilla, the systems of Altner and Prillinger [30] were used in addition to specialized nomenclature from other studies [31,32,33]. The terminology of mouthparts was adopted from Spangenberg et al. [34]. The terms dorsal, ventral, anterior, and posterior consistently refer to the longitudinal body axis (e.g., vertex dorsal, labium ventral), the mouthparts being considered as extending posteriad from the head capsule (opisthgnathous condition).

## 3. Results

### 3.1. Gross Morphology of the Mouthparts

The mouthparts in hemipteran/heteropteran taxa consist of a short, conical labrum and a long, segmented labium, bisected dorsally by a labial groove within which lie the mandibular and maxillary stylets. This functional complex is often called the “rostrum”, “beak”, or piercing–sucking mouthparts.

#### 3.1.1. Labrum

**Figure 1:**
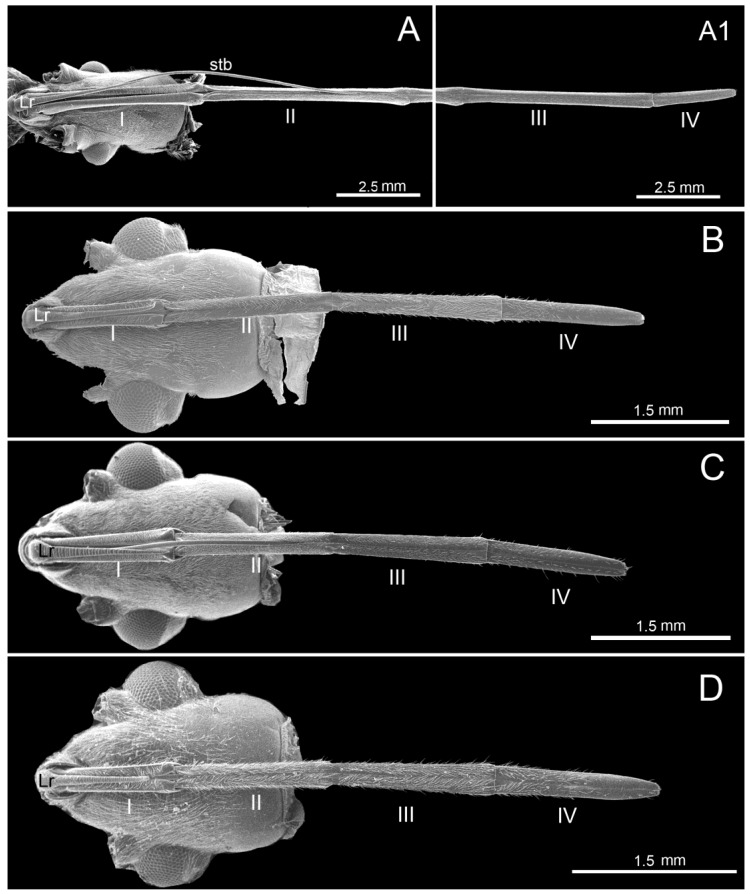
Scanning electron micrographs showing ventral view of the labium (segments I–IV), labrum (Lr), and stylet bundle (stb). (**A**) *Macrocheraia grandis* (Gray) (A1 showing III and IV segments); (**B**) *Physopelta quadriguttata* Bergroth; (**C**) *Physopelta gutta* (Burmeister); (**D**) *Physopelta cincticollis* Stål.

The elongated tongue-shaped labrum (Lr) was clearly separated from the anteclypeus (Figure 1A–D). The proximal part was wide, the distal part narrowed, and the surface was strongly plicated. The ventral side was densely covered with mechanosensilla trichodea (St1) in the studied largid species; sensilla chaetica (Sch) were not identified only in *Ph. quadriguttata*. In *Macrocheraia grandis* (Figure 1A), the labrum was 3999.6 µm long and covered the basal half of the first labial segment. In *Physopelta* species, lengths of the labrum were 1545.4 µm in *Physopelta gutta*, 1429.3 µm in *Ph. Quadriguttata*, and 1044.6 µm in *Ph. cincticallis*, and the labrum almost completely covered the first labial segment (Figure 1B–D).

#### 3.1.2. Labium

**Figure 2:**
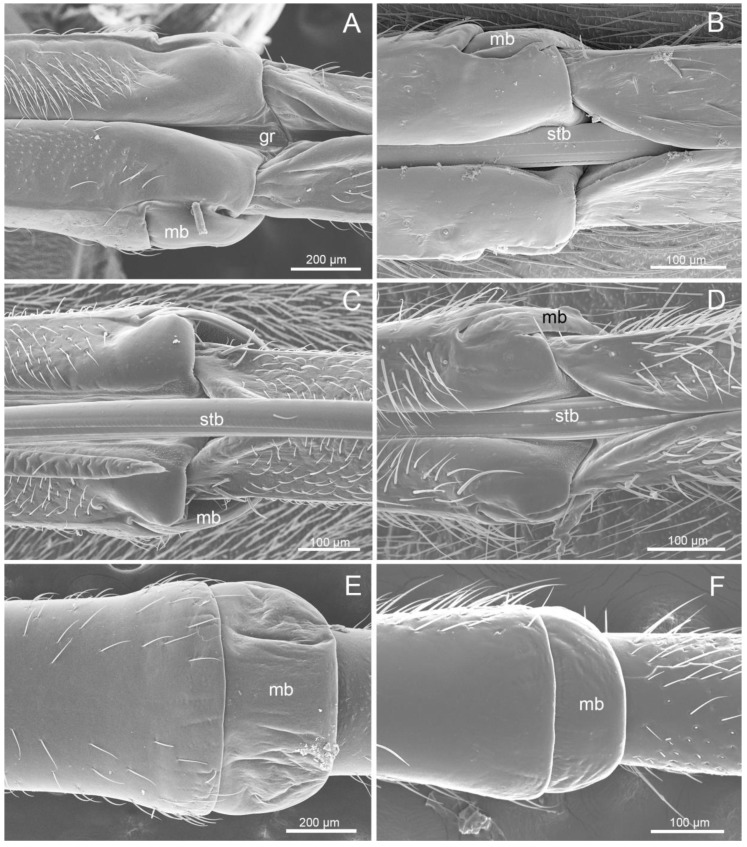
SEM images of the junction of the first and second segment. (**A**) Ventral view of *M. grandis*; (**B**) Ventral view of *Ph. quadriguttata.* (**C**) Ventral view of *Ph. gutta*. (**D**) Ventral view of *Ph. cincticollis*. (**E**) Dorsal view of *M. grandis*. (**F**) Dorsal view of *Ph. cincticollis*. stb: stylet bundle; gr: labial groove; mb: membrane.

The labium (Lb) of largids was tube-like and four-segmented, and formed a sheath for the mandibular (Md) and the maxillary (Mx) stylets (Figure 1A–D). In resting position, it was caudally oriented. In both tribes, the first (basal) segment (I) was strongly sclerotized, and was in contact with the stylets (stb) (Figure 1B–D), which were placed in a labial groove (gr) on its dorsal side. The labial groove passed through all four segments and it was wide in the first segment. In the second through fourth segments, the edges of the labial groove were closely appressed (Figure 2A–D). 

The first segment was of uniform width throughout most of its length, with the distal part widened. 

This part formed a distinct articulation with the second segment (Figure 2A–F) and consisted of a band-like dorsal plate (bdp) (sclerotized membrane) and ventro-lateral band of the membrane (mb) of the basal segment. These elements completely covered the joint between the two segments dorsally (Figure 2A–D) and ventro-laterally (Figure 2E,F). Externally, it was strongly sclerotized, whereas part of the dorsal band formed a membranous part.

The second and third segments were of similar width throughout most of their length, but they were narrowed in contrast to the first. A slight membranous extension from the ventral side was observed on the boundary between second and third segments. The fourth labial segment was conical and tapered distally (Figure 1A–D). The two last segments were separated by a distinct articulation of the membrane.

**Figure 3:**
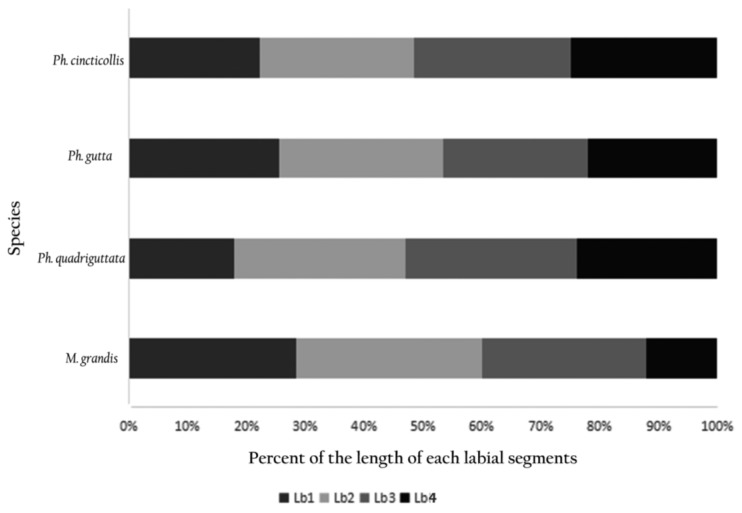
Percent of the length of each labial segment in different species. Lb1, 2, 3, and 4: the first, second, third, and fourth labial segments, respectively.

The first (I), second (II), third (III), and fourth segments (IV) had similar shapes but differ in length (Table 1). In *Macrocheraia grandis*, the three basal segments were approximately the same length (30% of each segment), and the last was the shortest (10%) (Figure 3). In *Physopelta cincticallis*, all segments were of similar length and in *Ph. gutta*, only the last segment was slightly shorter, about 20% of the total length of the labium. In *Ph. quadriguttata*, the second and third segments were the longest; the fourth was slightly shorter, and the first was the shortest (Table 1, Figure 3). The labium was the longest (19,203.9 µm) in *M. grandis* (Figure 1A) and shorter in *Physopelta* spp.: 7052.8 µm, 6712.6 µm, and 5414.6 µm (Table 1).

### 3.2. Labial Sensilla Types and Their Arrangement

**Figure 4:**
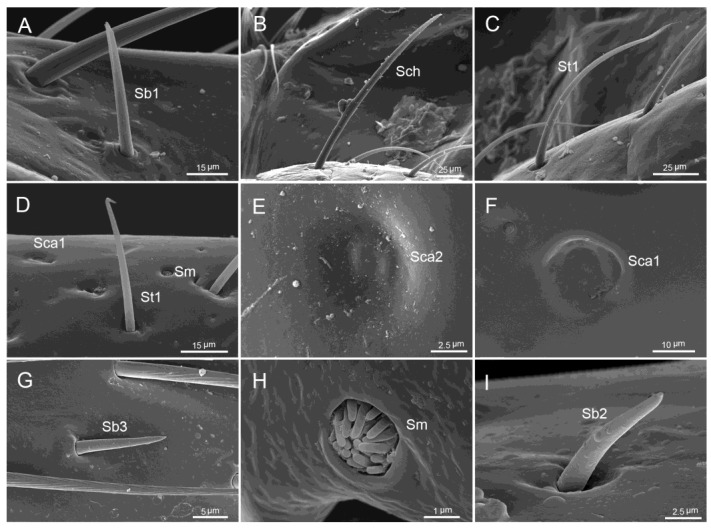
SEM images of sensilla of four species. (**A**) Sensilla basiconica1 (Sb1); (**B**) sensilla chaetica (Sch); (**C**) sensilla trichodea1 (St1). (**D**) Enlarged view of part of labium, showing sensilla trichodea1 (St1), sensilla campaniformia1 (Sca1), and sensilla multilobular (Sm); (**E**) sensilla campaniformia2 (Sca2); (**F**) sensilla campaniformia1 (Sca1); (**G**) sensilla basiconica3 (Sb3); (**H**) sensilla multilobular (Sm); (**I**) the view of sensilla basiconica2 (Sb2).

**Figure 5:**
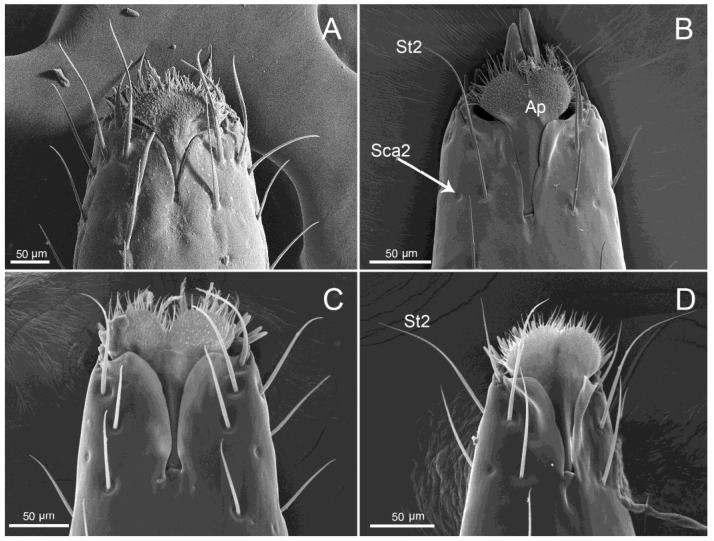
SEM images of labial tips of four species from a dorsal view. (**A**) *M. grandis*; (**B**) *Ph. Quadriguttata*; (**C**) *Ph. gutta*; (**D**) *Ph. cincticollis*. St2: sensilla trichodea2; Sca2: sensilla campaniformia2; Ap: apical plate.

**Figure 6:**
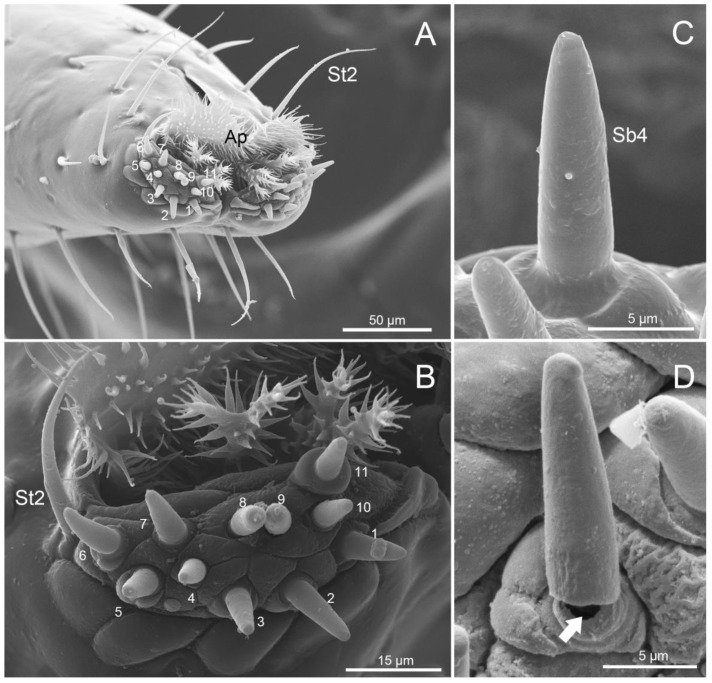
SEM images of the tip of the labium of *Ph. cincticollis*. (**A**) Vertical view of the tip of labium; Ap: apical plate. (**B**) Left side of labial tip showing sensilla basiconica4 (Sb4) (no. 1–10), sensillum styloconicum (no. 11) and sensillum trichodeum (St2). (**C**) Enlarged view of sensilla basiconica4 (Sb4). (**D**) Enlarged view showing hollow sensilla basiconica4 (Sb4).

Characteristics including the surface sculpture and pore system, socket form, and shape were used to classify sensilla (Table 2). Based on external morphology, 11 sub/types (subtypes based on the length and shapes were added to main types) of sensilla were observed on the surfaces of labial segments and the labial tip (Figure 4A–I, Figure 5A–D, Figure 6A–D, Table 3).

Sensilla types are categorized by their morphological characteristics as follows.

Sensilla chaetica are long and straight, with a blunt tip and a minute longitudinal groove in the shaft, and they are embedded in a cuticular sheath that forms a socket (Table 3, Figure 4B).

Sensilla trichodea were the most abundant sensilla observed in all species studied. These sensilla are hair-like with a curved and pointed apex. They are inserted in an elevation on the cuticle (flexible socket). Two subtypes were identified as St1 and St2 based on their lengths and external morphology (Table 3, Figure 4C,D, and Figure 6B). St1 have longitudinal grooves on the surface and St2 lack such grooves.

Sensilla basiconica are conical, straight, robust, relatively short, and with a smooth surface. Four subtypes were observed and categorized based on their external shape and size, presence of a flexible or inflexible socket and position as Sb1, Sb2, Sb3, and Sb4 (Table 3, Figure 4A,G,I, and Figure 6C). Sb1 are the longest and inserted in a circular depression. They were only present at the junctions of the first and second segment and the third and fourth segment. Sb2 are medium-sized pegs with a blunt tip. Sb3 are small pegs with tapered tips. Sb2 and Sb3 were inserted into flexible sockets and were present on the labium surface. Sb4 are cone-shaped sensilla with a pointed tip and are found at the center of the labial tip. Their structure and position suggest the gustatory function of these sensilla. 

Sensilla styloconica are robust, straight, with a smooth surface, and are inserted on a raised platform. These sensilla were present at the center of the labial tip (Figure 6B (no. 11)).

Sensilla campaniformia are cupola-shaped structures with the central part slightly convex. Two subtypes were categorized based on their size as Sca1 (large) and Sca2 (small) (Table 3, Figure 4E,F).

Sensilla multilobular contain several short cones, grouped inside a circular depression in the cuticle (Figure 4H).

The exposed surface of the labium was covered mainly with sparse, hair-like sensilla (basiconica, chaetica, trichodea) (Figure 1A–D, Figure 4A–C), dome-shaped sensilla campaniformia, and sensilla multilobular coeloconica (Figure 4E,F,H, Figure 5A–D).

Sensilla chaetica were sparse (low density) and visible on the second to third segments in *M. grandis*, on the second segment in *Ph.gutta*, and on the first and second segments of *Ph. cincticallis*, but absent in *Ph. quadriguttata*. Sensilla trichodea (St1) were more numerous and distributed on the first to fourth segments in all species. Several sensilla trichodea (St2) were distributed on the ventral side near the apical plate (Ap) in all species (Figure 5A–D). Sensilla basiconica (Sb1) were found only on the first and fourth segment in all species. Sensilla basiconica (Sb2) were distributed differently among species, occurring on the second and third segments of *M. grandis*, on third and fourth segments in *Physopelta gutta*, on the first and second segments in *Ph. quadriguttata* and only on the third segment in *Ph. cincticallis*. Sensilla basiconica (Sb3) were observed only in *Ph. quadriguttata*. Sensilla campaniformia (Sca1) were common and were usually found on the second segment in the studied species, while the other type (Sca2) was present on the fourth segment. Sensilla multilobular (Sm) were observed only in *M. grandis* on the first segment; however, we expect that they are also present in other species.

A specialized group of sensilla was present on the labial tip which is tripartite, consisting of two lobes and an apical plate (Figure 5A–D, Figure 6A). Sensilla were symmetrically arranged on the two lateral lobes forming two sensory fields (Figure 6A,B) each including three types of sensilla; short, stocky sensilla basiconica (Sb4, no.1–10); longer, stout sensilla styloconica (no. 11); and long, narrow sensilla trichodea (St2). Ten sensilla basiconica with a single terminal pore (Figure 6B,C) sat within a non-flexible socket and were located at the center of each lobe along with one multiporous sensillum styloconicum, while one long sensillum trichodeum (St2) was located behind the stylet groove near the apical plate. The sensilla basiconica (Sb4) slightly varied in size among the three species of *Physopelta* but these sensilla were distinctly longer in *M. grandis* (Table 3).

Generally, the apical plates of the largid bugs had a rostral lid, which possessed some membranous microtrichia (Figure 5A–D, Figure 6A). Two different shapes of apical plate were observed: cactoid undivided in *Macrocheraia grandis* (Figure 5A) and cactoid medially divided on the distal margin in the three species of *Physopelta* (Figure 5B–D).

### 3.3. Stylet Fascicle

The stylet bundle was strongly elongated, slender and consists of two mandibular stylets (Md) and two maxillary stylets (Mx) (Figure 1A). The latter were interlocked for almost their entire length, the ends of the bundle were usually slightly separated.

#### 3.3.1. Mandibles

The distal, external, and internal parts of the mandibular (Md) stylets were observed in the four largid species. The mandibular (Md) external serration formula gives the number of transverse ridges and teeth on the anterior and dorso-lateral side of the apex of mandibles (tr 7 + ct (1–2) + lt (1–2).

**Figure 7:**
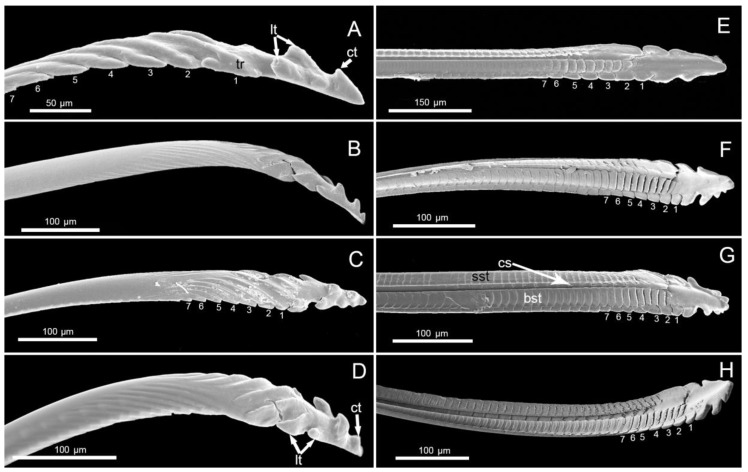
SEM images of mandibular stylets of four species. (**A**) Lateral view of stylet in *M. grandis* showing seven transverse ridges (tr), one central tooth (ct), and one pair of lateral teeth (lt). (**B**) Lateral view of of stylet of *Ph. quadriguttata*. (**C**) Lateral view of of stylet of *Ph. gutta.* (**D**) Lateral view of of stylet of *Ph. cincticollis* showing seven transverse ridges (tr), central tooth (ct), and two pairs of lateral teeth (lt). (**E**) Interior side of stylet of *M. grandis*. (**F**) Interior side of stylet of *Ph. quadriguttata*. (**G**) Interior side of stylet of *Ph. gutta* showing small squamous texture (sst), cutiular spines (cs), and bigger squamous texture (bst). (**H**) Interior side of stylet of *Ph. cincticollis.*

On the mandibular stylets of *Macrocheraia grandis* (Figure 7A), there were seven well-differentiated transverse ridges (tr). Apart from these ridges, there were several smaller ones. Counting from the apex, one narrow tooth (ct) was placed anteriorly and one pair of narrow teeth (lt) was arranged dorso-laterally. In the three species of *Physopelta* (*Ph. quadriguttata* (Figure 7B), *Ph. gutta* (Figure 7C), *Ph. cincticallis* (Figure 7D)), seven deep transverse ridges (tr) were visible and one or two short and narrow teeth (ct) were placed anteriorly. Two pairs of narrow teeth (lt) were placed dorso-laterally.

The inner surface of the mandibular stylet in the distal parts was heterogenous in structure and the same characters were present in all studied species. Dorsally there was one row of small squamous textures (sst), and among them there were cuticular spines (cs) of different length. The second row consisted of bigger squamous textures (bst) with different cuticular spines. Between them there was a longitudinal groove. From the inner side, the ends of the mandibles were smooth. The lateral teeth visible from the internal side of the end of the mandibles corresponded to the lateral teeth on the dorsal side. The dorsal surface had a row of serrate ridges and some scalelike projections were positioned on the lateral surface.

#### 3.3.2. Maxillae

**Figure 8:**
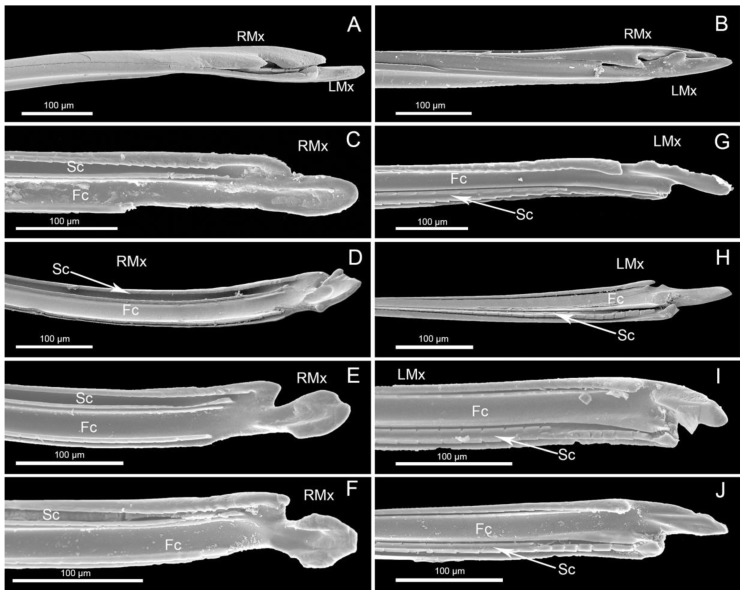
SEM images of maxillary stylets of four species. (**A**) *Ph. cincticollis*; (**B**) *Ph. gutta*. (**C**) Right maxillary stylet (RMx) of *M. grandis* showing food canal (Fc) and salivary canal (Sc); (**D**) *Ph. quadriguttata*; (**E**) *Ph. gutta*; (**F**) *Ph. cincticollis*. (**G**) Left maxillary stylet (LMx) of *M. grandis* showing food canal (Fc) and salivary canal (Sc); (**H**) *Ph. quadriguttata*; (**I**) *Ph. gutta*; (**J**) *Ph. cincticollis*.

The ends of the stylets are shown in Figure 8A–J. In largid species, the left–right asymmetry of the maxillary stylets was noticeable, and the ends of the maxillae had different shapes (Figure 8A,B).

In the studied taxa, the apex of the left maxilla (LMx) was more sharply pointed than that of the right maxilla (RMx) (Figure 8A–J). The apex of the right maxilla was tapered in *M. grandis* (Figure 8C), but flattened and spoonlike in *Ph. quadriguttata* (Figure 8D), *Ph. gutta* (Figure 8E), and *Ph. cincticallis* (Figure 8F)). The apex of the left maxilla was straight and narrow in *M. grandis* (Figure 8G), while in the three other species (*Ph. quadriguttata* (Figure 8H), *Ph. gutta* (Figure 8I), and *Ph. cincticallis* (Figure 8J)), it was straight and distinctly wider. In all studied species, the anterior and posterior margins of the left maxilla had distinct incisions corresponding to the protruding parts of the margins on the right maxilla. On the inner sides of both stylets were visible ridges of the salivary and food canals, which were terminated before the narrowed apex (Figure 8C–J). The ends of each stylet and the anterior (ventral) and posterior (dorsal) margins on the external surface were smooth.

#### 3.3.3. Cross-Section of the Stylet Bundle

**Figure 9:**
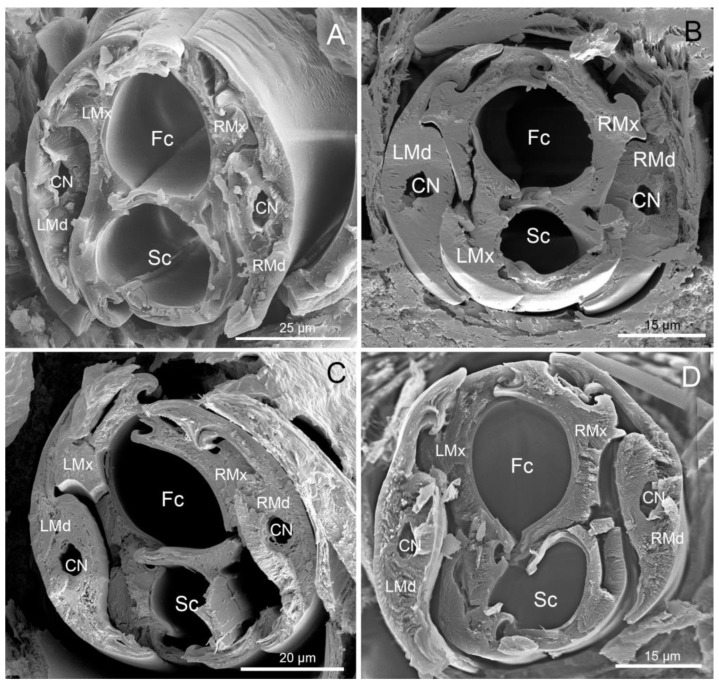
Cross-section of stylet of four species showing nerve canal (CN), food canal (Fc), salivary canal (Sc), left mandibular stylet (LMd), left maxillary stylet (LMx), right mandibular stylet (RMd) and right maxillary stylet (RMx). (**A**) *M. grandis* (Gray); (**B**) *Ph. quadriguttata*; (**C**) *Ph. gutta*; (**D**) *Ph. cincticollis*.

**Figure 10:**
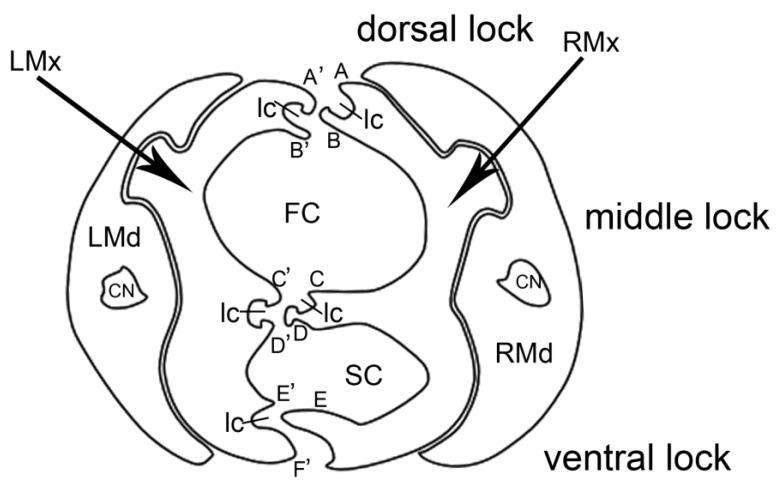
Cross-section of stylet fascicle of Largidae. A. Diagram of cross-section of stylet fascicle. LMd: left mandibular stylet; RMd: right mandibular stylet; LMx: left maxillary stylet; RMx: right maxillary stylet; Fc: food canal; Sc: salivary canal; Ic: interlocking canal; CN: dendritic canal; A: straight lock; A’: hooked lock; B: hooked lock; B’: straight lock; C: straight lock; C’: hooked lock; D: T-shaped lock; D’: hooked lock; E: hooked lock; E’: hooked lock; F: straight lock.

Cross-sections (Figure 9A–D) showed that the stylet bundle was distinctly laterally compressed, taller than it was wide. The two maxillae were held together by interlocking processes forming three locks: dorsal, median, and ventral. The dorsal lock had two hooked processes and two straight processes. The middle lock had two hooked processes, one straight process and one T-shaped. The ventral lock had one straight and two hooked processes (Figure 10). Inside, the locked maxillary stylets formed a salivary canal (Sc) and a food canal (Fc), which are used for delivering saliva to the plant and to suck plant fluids, respectively. The hollow food canal was ovoid, slightly greater than the salivary canal, and was mostly located in the right maxilla. The two mandibular stylets were mirror images of each other, and connected by a one-lock system with maxillary stylets. Within each mandibular stylet, there was one approximately semicircular dendritic canal (Figure 10).

## 4. Discussion

This study presents detailed observations of the mouthpart structures in Largidae (Heteroptera: Pyrrhocoroidea). These reveal some new and interesting features that differ between species of the tribes Physopeltini and Lohitini, and allow comparison of and better understanding of the feeding strategies and the sensory systems of these bugs (mainly seed-sucking) compared to other heteropterans. 

### 4.1. Mouthpart Morphology

There were distinct differences in total length of the labium among the four largid bug species observed (longest in *Macrocheraia grandis* (Lohitini: 19,204 µm), being shorter in Physopeltini (7053 µm; 6713 µm; 5415 µm, Table 2)). In *Pyrrhocoris sibiricus*, the labium was much shorter (3487 µm) [17]. The mentioned species belong to two closely related families of seed-feeding bugs, so the differences in length of the labium may be regarded as taxonomic characteristics for these families. Moreover, the length of the labium may also be useful for distinguishing largid tribes.

Differences of the labium length have been reported in four chinch bug species (Blissidae), which feed on the sap (rather than the seeds) of plants, but the author suggested that these parameters are unlikely to be major factors in the chinch bugs’ ability to feed on different plants [36]. This is in accordance with some data suggesting that the length of the labium plays an important but indirect role in feeding [17]. Generally, in phytophagous pentatomomorphan taxa, the second segment usually bends toward the insect’s body during feeding, shortening the functional length of the labium and allowing for deep penetration of the stylets [37]. If the insertion is successful, the labium continues to curve to a maximum angle between the first and the second segments. In these bugs, shortening of the labium during feeding by deflecting the second segment is facilitated by the wide membranous connection between the first and second segment. This structure is present in the studied largid species, and it has also been observed in pyrrhocorids [17].

The mandibular stylets were minimally serrated and their serration formulae were very similar among the studied taxa. *Physopelta* species had one additional anterior tooth and one additional pair of dorso-lateral teeth compared to *Macrocheraia*. These mandibular structures also differed from those of *Pyrrhocoris sibiricus* (three central teeth and two pairs of lateral teeth) [17]. In other phytophagous species such as *Dysdercus fasciatus* Sign [38] or *Odontopus nigricornis* Stål [33], nonsignificant differences in numbers and shape of the teeth have been observed compared to the studied largids. The small differences in mandibular serration among species of Largidae and Pyrrhocoridae appear to reflect their close phylogenetic relationship and similar feeding behavior. Almost the same serration pattern of mandibles is present in both largid tribes and related families (7s+ 1–3 ct+ 1–2 lt). A significant number of teeth (from 1 to 20) are present on the mandibles in other pentatomomorphan families, coreoids and lygaeids [8], although the latter are also seed-feeders. The well-formed mandibular teeth in the mentioned taxa are used in lacerate-and-flush feeding and serve to pierce the plant tissues and then anchor the mandibles in the tissue [8,39,40,41,42]. The inner surface of the mandibles of each largid species has a complex ribbed squamous texture. According to Cobben [8] and Wang and Dai [17], such structures exists in many other plant-feeding species and produce considerable friction with the outer surface of the adjacent maxillary stylets, causing it to bend inward when probing plant tissue.

In all predatory groups, the mandibular stylets have a special dentition (spines) that produces a penetrating, tearing, or filing device that aids in the mechanical disruption of host tissues [8], and which is evidently more developed than in the phytophagous species of these bugs.

Usually, the tips of the maxillary stylets are sharply pointed in heteropteran species; however, they are modified in different ways in different taxa [12,15,43], depending on the food source.

The present analysis of the maxillar stylets showed one essential difference between *Physopelta* and *Macrocheraia*. The asymmetrical shape of the maxillar apex in *Physopelta* (right tip spoon-like and left tip straight and narrow) is probably a modification related to seed-feeding. In contrast to *Physopelta*, more narrowed ends of the maxillae were characteristic of *Macrocheraia*. However, in both taxa, the distal end of each maxilla was smooth. Images of the maxillae of related pyrrhocorid species (*Dysdercus fasciatus* or *Pyrrhocoris sibiricus*) have been published previously [17,38]. In these cases, the distal end of each stylet is flat and there are very small spines on the anterior and posterior edges of the external surfaces [17,38]. Although these differences are minimal, they represent differences among species of related taxa, most of which are typical seed-sucking insects that occasionally also suck sap. In contrast, in some taxa of predaceous heteropterans, the distal parts of the maxillary stylets have different types of barbs consisting of rows of very well developed stiff bristles [8,15].

We did not observe any differences in the cross-sections through the tips of the stylet bundle among largid species. All typical interlocking structures of the stylets and proportions between food and salivary canals were similar to those of other pentatomomorphan species [16,44,45].

### 4.2. Adaptation to Seed-Feeding

In insects, seed-sucking species are generally morphologically and physiologically adapted to use seeds as a resource and are generally specialists in tissue and plants [46].

Our observations on *Physopelta* species suggest they are specialized for seed-feeding. Their strong mandibular teeth and asymmetrical maxillae facilitate a lacerate-and-flush feeding method with the ability to feed also on soft tissues of plants (fruits). *Macrocheraia* appears to be more adapted for phloem feeding, based on the long and more narrowed end of the maxillary stylets and enough short teeth to anchor the stylets in plant tissue. Backus [13] reported the feeding strategy of seed-sucking Pentatomidae (Pentatomomorpha), and noted that each mandible has teeth at the distal end that tear the feeding substrate and allow attachment of the maxillary stylets and stationary feeding. However, observations of seed-sucking coreoids [2,47] showed another strategy in which the stylets move back and forth continuously from a given perforation point, and a similar mechanism of penetration was described by Wang and Dai [17] in *Pyrrhocoris sibiricus*. Most seed-sucking insects are able to use other food sources without seeds. Even when seeds are plentiful, they use other sources to get water [20,47].

Prior to feeding, various Heteroptera detect the surface of seed by expelling wet saliva, which is sucked back again then rubbed back and forth across the tip of the rostrum [48]. Such behavior supports the idea that the sensilla of the labial tip provide essential information about the chemical composition of the substrate. Furthermore, several researchers [33,35,49,50,51,52] have determined that the apical labial sensilla receptors of feeding and seed-sucking insects are important chemical and mechanical receptors in host selection and feeding in hemipteran species.

### 4.3. Sensilla Types

Prior to this, detailed morphological of largid mouthpart sensilla had not been studied. The tip of the labium of largid species bears a set of 12 sensilla consisting of three types, which presumably have gustatory (Sb4, 10), olfactory/gustatory (SCo, 1), and tactile (St2, 1) functions.

Comparisons of the labial tip sensilla of the studied largid species with species of Pyrrhocoridae (*Pyrrhocoris sibiricus* by Wang and Dai [17], *Dysdercus fulvoniger* and *D. koenigii* by Schoonhoven and Henstra [53], *D. fasciatus* by Peregrine [29], and *D. intermedius* by Gaffal [39]) have revealed significant variation in structure and arrangement. In *Dysdercus*, sensillum basiconicum D may be an intermediate form between a single-walled orifice olfactory sensillum and a terminal-pore taste receptor. This sensillum may be homologous to the sensillum styloconicum (SCo) in largid bugs; it is absent in *P. sibiricus*, but in this species, one pair of sensilla basiconica with perforated walls was observed. The labial tip sensilla in pyrrhocorids and largids usually consist of 11 to 12 sensilla basiconica (Sb4) and one sensilla trichodea (St2), except in *Dysdercus fasciatus* [29] and *Odontopus nigricornis* Stål [33], with 10 sensilla basiconica. Significantly more numerous sensilla basiconica, ranging from 16 to 22, have been reported in other species of pentatomomorphans (in the alydid *Riptortus pedestris* F. and the lygaeid *Elasmolomus sordidus* (F.) [35]; in the pentatomid *Nezara viridula* L. [33], and in *Blissus leucopterus* (Say)) [54]. Sensilla basiconica or peg sensilla usually have the same function (gustatory) on the labial tip, but they differ in quantity among the mentioned species.

Previous literature has not reported the presence of typical multiporous olfactory sensilla on the labium in Pentatomomorpha. However, single-pore sensilla that respond to taste stimuli can also respond to strong odors [55], and olfactory stimuli may thus be detected by the labial tip. Moreover, in some other hemipterans, typical multiporous olfactory sensilla have been observed on the labial tip [56,57,58].

Sensilla trichodea on the apical and subapical region of the labium in largids probably represent mechano-chemosensilla. Their appearance and location were similar to those reported as having this dual function in other insects [30,50,59], as well as in phytophagous and predatory species of Pentatomidae [29,33,52,60,61]. Generally, the labial tip sensilla in heteropteran insects, although more or less numerous and of different shapes and sizes, have been regarded as morphologically analogous by previous authors [8,33,39,53,56,62,63,64,65,66].

The labium surface is usually equipped with a large group of different types of mechanosensilla and a smaller group of thermo-hygrosensitive sensilla. Our observations of mechanosensilla on the labial surface in largids showed that the number, types, and distribution were similar among species. Only in *Ph. quadriguttata* were sensilla chaetica not observed. In the remaining largids, sensilla chaetica were not numerous but were present singularly or in small groups on the first to third segments. The long sensilla trichodea (St1) with flexible sockets covering almost the whole surface of the labium and with structure indicating a tactile function appeared to be identical to those of pyrrhocorids [17] and other heteropterans [29,33,52,61]. The pair of sensilla basiconica (Sb1) observed in largid species, present on the junction between the first and second segments and between the third and fourth segments, are proprioceptors that perceive the degree of flexion of the joint of these segments. Such proprioceptive sensilla occur in many hemipteran and heteropteran taxa [17,67]. The arrangement of sensilla basiconica (Sb2) on the anterior surface of the fourth segment in largids differs depending on the species. These sensilla correspond to common type of sensilla basiconica also observed in other Hemiptera [57,68]. Very small sensilla basiconica (Sb3) with tapered tips were found on the third labial segment only in *Ph. quadriguttata*. However, similar sensilla basiconica (Sb3) were found on the fourth segment in most previously studied pentatomonorphans (*Pyrrhocoris sibiricus* [17], *Odontopus nigricornis*, and *Nezara viridula* L. [33]; and in *Riptortus pedestris* F., *Elasmolomus sordidus* (F.), *Cyclopelta siccifolia* Westwood, and *Chrysocoris purpurea* (Westwood) [35].

Sensilla campaniformia (SCa) are widespread on the insect body and frequently occur on the mouthparts, especially in areas that undergo deep deformation or stretching [51,65], such as on the bases of wings, halteres, legs, and antennae [69,70]. In largids, sensilla campaniformia (Sca1) are not numerous and occur sporadically on the first to third labial segments, similarly to the very small sensilla campaniformia (Sca2) present only on the fourth segment. Sensilla campaniformia (two pairs) are also present on the distal part of the anterior surface of the second labial segment of *P. sibiricus* [17]. In the studied species, such sensilla probably act as proprioceptors responding to the stresses arising from the movement of the labium. A separate group of sensilla on the labial surface are thermo-hygrosensitive sensilla. Among largid bugs, sensilla multilobular were observed only in *M. grandis*; they corresponded to sensilla coeloconica situated on the surface of the labium in other heteropteran species. Our study could not rule out the presence of such sensilla in *Physopelta* because their very small size made them difficult to observe and identify using SEM. Previous studies of heteropterans/hemipterans indicate that morphologically similar sensilla are present in most species [32,50,56,58,66,71].

In most heteropteran taxa, the apical plate of the labium is relatively preserved in different shapes [2,8,17,32,38,52,72]. We found two forms of the apical plate in largid species. In *Physopelta* the apical plate was partly divided on the distal margin, in contrast to *Macrocheraia grandis*. A similar undivided cactoid apical plate was observed in *Pyrrhocoris sibiricus* [17] as well as in *Dysdercus* species [73]. Additional structures connected with the apical plate have been discussed by several authors [8,61,74], who concluded that the plate does not function in any sensory capacity but may have a mechanical function, recording friction between stylets during sucking and providing better control over the movements of the rostrum.

## 5. Conclusions

The mouthpart structures of four Largidae species were investigated as examples of seed-feeding heteropterans. Our results represent the first detailed reports of *Macrocheraia* and *Physopelta* species mouthpart structures. Compared with other Pyrrhocoridae, the mouthparts of the studied taxa appear to display a number of traits that are evidently common in both families: similar labium shape but different lengths, identical types of sensilla on the labial surface, and almost identical apical plates. Slight differences were observed between the Physopeltini and Lohitini tribes in the tips of the mandibular and maxillary stylets (different serration on the mandibular apex and shapes of the maxillar end in different taxa). Sensilla of the labial tip in largids were highly similar in structure and arrangement, but not identical to those of the pyrrhocorids *P. sibiricus* and *Dysdercus*. Although the mouthparts have evolved in ways that allowed these insects to effectively exploit identical food sources, some structures of their mouthparts represent distinct characteristics. Generally, the structure of the mouthparts is slightly different between seed-feeding and sap-feeding species. However, almost identical functional components are found in all groups of Pentatomomorpha; these differ from mouthparts of the predatory bugs from which they apparently evolved.

## Figures and Tables

**Table 1:** Comparison of the lengths of labial segments in studied species. Data are means ± SE values obtained from scanning electron microscopy.

Species	Segment				
	I (μm)	II (μm)	III (μm)	IV (μm)	Whole
*Macrocheraia grandis* (Gray)	5451.1 ± 59.9	6069.9 ± 112.2	5352.3 ± 11.8	2330.5 ± 34.5	19,203.9 ± 122.3
*Physopelta gutta* (Burmeister)	1767.4 ± 61.1	1940.2 ± 26.3	1706.0 ± 56.8	1527.5 ± 7.4	7052.8 ± 68.1
*Physopelta quadriguttata* Bergroth	1494.3 ± 12.4	1807.8 ± 6.4	1795.6 ± 17.18	1475.6 ± 18.1	6712.6 ± 117.6
*Physopelta cincticollis* Stål	1191.6 ± 60.1	1420.1 ± 80.5	1432.6 ± 84.5	1343.8 ± 50.2	5414.6 ± 151.1

**Table 2:** Terminology and definition of sensilla used in the present paper [30,31,32,35].

Category	Function	Pore	Sensilla Type
Mechanoreceptive sensilla	Tactile	NP: no pore	Sensilla chaetica or sensilla trichodea: sharp tip haired sensilla in a basal flexible socket; sensilla basiconica (Sb2, Sb3): tapered tip haired sensilla in a basal flexible socket; sensilla campaniformia (Sca1, Sca2): dome shaped positioned at or below cuticular surface
Chemoreceptive sensilla	Gustatory Olfactory/gustatory	UP: uniporous with one terminal pore MP: multiporous	Sensilla basiconica 4 with an inflexible basal socket, Sensillum styloconicum: cone sitting on a style (high socket)
Thermo-hygroreceptive sensilla	Temperature/humidity	NP: no pore	Sensilla multilobular (Sm): pegs in cavity surrounded by fingerlike structures, no flexible sockets
Proprioceptive sensilla	Perceive the degree of flexion of the joint	NP: no pore	Sensilla basiconica 1: on the junction between the first and second segment, and the third and fourth segment

**Table 3:** Distribution and morphometric data of sensilla in studied species. Data are means ± SE values obtained from scanning electron microscopy. N = sample number; Lr: labrum; Sch: sensilla chaetica; Str1-2: sensilla trichodea I-II; Sb1-4: sensilla basiconica I-IV; Sst: sensilla styloconica; Sca1-2: sensilla campaniformia I-II; Sm: sensilla multilobular; Lb: labium; Lb1, 2, 3, 4: the first, second, third, fourth segment of labium; SF: sensory field on the labial tip.

Sensilla	*Macrocheraia grandis* (Gray)				*Physopelta quadriguttata* Bergroth				*Physopelta gutta* (Burmeister)				*Physopelta cincticollis* Stål			
	Distri-bution	Length (μm)	Basal Diameter (μm)	N	Distri-bution	Length (μm)	Basal Diameter (μm)	N	Distri-bution	Length (μm)	Basal Diameter (μm)	N	Distri-bution	Length (μm)	Basal Diameter (μm)	N
Sch	Lr, Lb2,3	183.4 ± 1.8	7.7 ± 0.3	5					Lb2	56.9 ± 0.2	7.9 ± 0.2	4	Lb1,2,	114.4 ± 6.1	4.7 ± 0.2	5
St1	Lr, Lb	71.9 ± 4.8	3.6 ± 0.2	9	Lr, Lb	57.3 ± 2.6	2.4 ± 0.1	10	Lr, Lb	39.4 ± 3.4	2.7 ± 0.1	6	Lr, Lb	60.1 ± 3.3	2.3 ± 0.3	5
St2	Lb4	119.6 ± 9.7	5.0 ± 0.2	11	Lb4	71.8 ± 5.7	3.2 ± 0.2	10	Lb4	65.3 ± 4.5	4.2 ± 0.4	6	Lb4	85.3 ± 4.1	3.5 ± 0.2	10
Sb1	Lb1,4	40.5 ± 4.8	4.9 ± 0.2	6	Lb1,4	24.6 ± 2.2	4.2 ± 0.1	7	Lb1,4	31.6 ± 0.7	4.5 ± 0.2	7	Lb1,4	28.5 ± 2.0	3.6 ± 0.2	10
Sb2	Lb2,3	21.0 ± 1.8	2.4 ± 0.1	3	Lb1,2	10.1 ± 0.5	1.9 ± 0.1	3	Lb3,4	12.0 ± 0.7	1.9 ± 0.1	3	Lb3	13.9 ± 0.8	1.8 ± 0.1	4
Sb3					Lb3	13.3 ± 0.1	1.8 ± 0.1	3								
Sb4	SF	19.0 ± 0.4	5.1 ± 0.2	10	SF	15.7 ± 0.6	4.2 ± 0.1	10	SF	15.1 ± 0.8	4.5 ± 0.1	16	SF	14.0 ± 0.4	3.5 ± 0.1	20
Sst	SF	18.8 ± 1.1	6.0 ± 0.1	3	SF	16.2 ± 0.3	3.6 ± 0.1	4	SF	16.0 ± 0.5	4.6 ± 0.1	6	SF	13.2 ± 0.4	4.0 ± 0.1	4
Sca1	Lb1, 2		14.3 ± 0.6	3	Lr, Lb2		6.4 ± 0.5	6	Lb 2		14.5 ± 0.6	6	Lb2		13.6 ± 0.4	4
Sca2	Lb4		5.5 ± 0.7	3	Lb4		4.9 ± 0.5	6	Lb4		5.2 ± 0.4	6	Lb4		2.9 ± 0.1	3
Sm	Lb1		2.8 ± 0.2	3												
